# Exploring the Epigenetic and Metabolic Pathways for Antioxidant and Anti-Inflammatory Potentials of Tart Cherry Juice Concentrate

**DOI:** 10.1007/s40495-025-00422-1

**Published:** 2025-07-26

**Authors:** Jiawei Xu, Yuxin Pan, Rebecca Mary Peter, Pochung Jordan Chou, Parv Dushyant Dave, Ahmad Shanner, Md. Shahid Sarwar, Lugui Brunetti, James E. Simon, Ah-Ng Tony Kong

**Affiliations:** 1 Department of Pharmaceutics, Ernest Mario School of Pharmacy, The State University of New Jersey, Rutgers, Piscataway, NJ 08854 USA; 2 Graduate Program of Pharmaceutical Sciences, Ernest Mario School of Pharmacy, Rutgers, The State University of New Jersey, Piscataway, NJ 08854 USA; 3 Department of Pharmacy, Noakhali Science and Technology University, Noakhali, 3814 Bangladesh; 4 Department of Pharmacy Practice, Ernest Mario School of Pharmacy, Rutgers, The State University of New Jersey, Piscataway, NJ 08854 USA; 5 Plant Biology and Pathology, Rutgers, The State University of New Jersey, New Brunswick, NJ 08901 USA

**Keywords:** Tart Cherry, Phytochemicals, Metabolism, Antioxidant, Anti-inflammation, Anthocyanins, Vitamin C, Beta-Carotene, Ellagic Acid, Chlorogenic Acid

## Abstract

Tart cherry (TC; *Prunus cerasus*) has high antioxidant and anti-inflammatory potentials due to its rich bioactive components like anthocyanins, polyphenols, vitamins, beta-carotene, ellagic acid, and chlorogenic acid. Oxidative damage and inflammation are underlying reasons to chronic disease pathogenesis. Oxidative stress usually caused by the imbalance between antioxidants and pro-oxidants. Additionally, a chronic inflammatory state is typically modulated by oxidative stress. Inflammation plays a critical role in chronic health conditions, such as cardiovascular diseases, hypertension, insulin resistance, arthritis and cancer. Numerous studies indicate that there is a strong relationship between TC and the inhibition of inflammation and oxidative damage by regulating different epigenetic and metabolic pathways. In this review, the recent developments of TC components and their metabolites on inflammatory and oxidative damages will be discussed, and the challenges and limitations to better support future research, including clinical trials to confirm these findings.

**Graphical Abstract:**

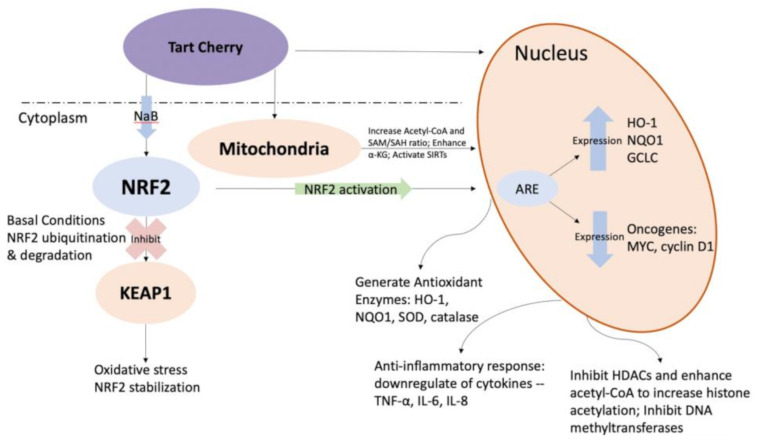

## Introduction

Inflammation and oxidative stress are common drivers of many chronic diseases, like cardiovascular diseases (CVD), diabetes, and cancer. Inflammation can functionally induce abnormal cell proliferation, attract inflammatory cells, and increase the generation of reactive oxygen species (ROS) within cells. Those changes can lead to DNA damage and hinder DNA repair mechanisms in inflamed tissues and organs. When cells are exposed to sustained inflammatory factors, chronic diseases will start to evolve [1]. Furthermore, the increase of ROS leads to oxidative stress and changes of protein modification [2, 3]. Then, protein oxidation can induce the generation of inflammatory signals such as peroxiredoxin 2 (PRDX2) [4]. PRDX2, as a redox-active enzyme, can activate macrophages to release cytokine, which leads to enhanced inflammation and chronic diseases [3].

Tart cherry (TC; *Prunus cerasus*) products, according to the American Botanical Council, were one of the most purchased botanical dietary supplements (BDS) by consumers in 2022 [5]. The effects of many of these products on human health are not fully understood. TC is believed to possess potent antioxidant and anti-inflammatory properties that may help with chronic conditions [6]. Several TC clinical trials have been reported interrogating its role in exercise recovery [7, 8], cardiovascular health [9–11], and gout [12], but all suffer from deficiencies or gaps that prevent the generalizability of their findings. These include a lack of information on the active constituent(s) essential for efficacy, failure to utilize pharmacokinetics (PK) and pharmacodynamics (PD) for dose optimization, inadequate identification of appropriate PD markers, and a lack of integration of data from preclinical testing into trial design. A table (Table 1) was made for concluding some clinical studies with TC, which summarizing the methodologies and results of key clinical studies of TC juice.

**Table 1:** Methodologies and results of key clinical studies of TC juice

Study Design	Sample Size & Population	Intervention	Primary Outcomes Measured	Key Result
Parallel, randomized controlled trial (PCT, RCT)	37 men and women between the ages of 65–80	Subjects were randomly assigned to consume either 480 mL tart cherry juice or control drink daily for 12 weeks	Increased the plasma levels of DNA repair activity of 8-oxoguanine glycosylase; Lowered the mean c-reactive protein (CRP) level	Verified the ability to reduce systolic BP and LDL cholesterol [13]
RCT	SPF-grade male Sprague Dawley rats 5 groups (*n* = 9)	Model group, positive group, and two experimental groups: adenine with 5% sodium carboxymethylcellulose suspension (50 mg/kg·bw) and oteracil potassium (1.50 g/kg·bw) with 5% sodium carboxymethylcellulose suspension; Control group: 5% sodium carboxymethylcellulose solution; After 5 h, control group and model group: pure water; Positive group: allopurinol solution (27.0 mg/kg·bw); Two experimental groups: TC powder suspension (0.17 g/kg·bw and 0.50 g/kg·bw); Reagents were administrated by gavage at a volume of 5 mL/kg·bw once a day for continuously 45 days	Low dose of TC powder: slightly decreasing serum uric acid and improving kidney injury; High dose of TC powder: merely alleviate kidney injury	Low dose of TC powder: beneficial to hyperuricemia through reduction of ADA activity [14]
PCT, 20-day, parallel, single-blind, placebo-controlled trial	45 participants, eighteen years of age and above, non-smoker, BMI < 30	Subjects were assigned to receive 60 mL per day of either Montmorency tart cherry juice, blueberry juice or a cherry/blueberry flavored placebo	Primary outcome: the between-group difference in systolic blood pressure from baseline to post-intervention; Secondary outcome: between-group differences in anthropometric, energy expenditure and substrate oxidation during rest and physical activity, haematological, blood pressure/resting heart rate, psychological wellbeing, and sleep efficacy indices	Explored the effects of both Montmorency tart cherry and blueberry juice on the primary and secondary outcomes pertinent to the aetiology of cardiometabolic disease and its comorbidities [15]
Randomized, single-blind, placebo-controlled, parallel-arm pilot clinical trial	19 men and women 20 to 60 years of age (Tart Cherry; *n* = 5 males, 4 females; Control; *n* = 5 males, 5 females)	240 mL of tart cherry juice for Tart Cherry group; 240 mL of isocaloric placebo-control drink for Control group; Twice daily for 12 weeks	Oxidized low-density lipoprotein and soluble vascular cell adhesion molecule-1 were significantly lower in TC than Control at 12 weeks; There was a trend for total cholesterol to be lower in TC than Control at 12 weeks	Daily tart cherry consumption may attenuate processes involved in accelerated atherogenesis without affecting hemodynamics or arterial stiffness parameters in this population [16]
Randomized, single-blind, placebo-controlled, crossover trial	12 (6 males and 6 post-menopausal females) participants, age 50 ± 10 years (range 28–62 years), body mass 94.1 ± 23.1 kg with MetS	Participants with MetS consumed montmorency tart cherry juice (MTCJ) or placebo (PLA) for 7 days	24-h ambulatory systolic, diastolic blood pressure and mean arterial pressure were significantly lower after 7-days for MTCJ group; Glucose, total cholesterol, LDL concentrations, total cholesterol: HDL ratio and respiratory exchange ratio values were significantly lower after 6-days of MTCJ group	Responses demonstrated clinically relevant improvements on aspects of cardio-metabolic function, emphasising the potential efficacy of MTCJ in preventing further cardio-metabolic dysregulation in participants with MetS [17]

TC contains phytochemicals like anthocyanins, polyphenols, vitamins, ellagic acid, chlorogenic acid, and carotenoids, which possess significant antioxidant activity and free radical scavenging effects [6, 17]. Anthocyanin is a water-soluble component in TC juice which is mainly absorbed in the stomach and small intestine in prototype form, and excreted through kidney after transformation [18, 19]. Anthocyanins can be found in many plants and dark color fruits, the therapeutic effects are confirmed on inflammation related diseases [19]. Anthocyanins can scavenge free radicals as strong antioxidants, and reduce the certain blood biomarkers to regulate blood lipid condition decreasing the risk of CVD [20]. Based on previous studies, anthocyanins scavenge free radicals through two pathways. The first one involves the attack on the hydroxyl group(s) of the B-ring in anthocyanin structure, while the second involves the attack on the oxonium ion of the C-ring [20]. Toll like receptors (TLRs) are a kind of innate immune receptors, usually mediate endotoxin reaction caused by lipopolysaccharide (LPS), and regulate TLR4/CD14 inflammatory signaling pathways. TLR4 receptor can activate the NF-κB signaling pathway by binding to a ligand LPS and then induce the expression of inflammatory factors like TNF-α, IL-6, IL-1β, and COX-2 [19]. Anthocyanins can downregulate inflammatory gene expression to control the transactivation of relative transcription factor [21, 22].

This review aims to explore the potential role of TC juice concentrate in influencing epigenetic changes and metabolic pathways, with an emphasis on understanding the underlying mechanisms and their implications for health and disease management. And synthesize current knowledge to guide future research and practical applications of TC in health and nutrition.

## Epigenetic and Metabolic Regulation by TC Components

### Anthocyanins

Anthocyanins are water-soluble flavonoids emitting red, blue, and purple colors in vegetables [23]. Natural dietary sources of anthocyanins include berries, grapes, cabbages, plums, and vegetables/ fruits with high red, blue, and purple pigment levels. Six typical anthocyanidins containing cyanidin, delphinidin, malvidin, peonidin, petunidin, and pelargonidin are derived from a flavylium cation backbone with hydroxylated modification on different carbons [24]. Enzymatic glycosylation of anthocyanidins via glucosyltransferase to yield the corresponding anthocyanins can take place on various hydroxyl groups of the molecule, with 3-OH being the most prevalent glycosylation site in nature. This process results in the formation of 3-*O-β*-glucosides [25]. The color and stability of anthocyanins rely on pH, temperature, structure, and the presence of light, oxygen, and metal ions [20]. In research, anthocyanins exhibit beneficial effects in mitigating the progression of cancer and metabolic syndromes through their antioxidant, anti-inflammatory, and epigenetic properties [26].

TC juice has been extensively studied for its potential to alleviate inflammatory conditions, particularly gout. Anthocyanins possessing anti-inflammatory and antioxidant properties are vital components and contribute to the efficacy in managing these diseases [27, 28]. The majority of anthocyanins in cherry are cyanidin compounds [29]. In TC and its products, the most abundant anthocyanin is cyanidin-3-glucosyl-rutinoside (C3GR), followed by cyanidin-3-rutinoside (C3R), cyanidin-3-sophoroside and cyanidin-3-glucoside [30]. Another study also claims cyanidin-3-glucosyl-rutinoside is the most content in TC juice and human plasma followed by cyanidin-3-rutinoside, which is 343.3 and 143.4 ng µL − 1, respectively [28, 31].

Persico et al.’s research demonstrates that a ten-month corn matrix enriched with C3G, when consumed by C57BL/6J female mice, induces a remodeling of H3K4me3 within the liver chromatin. This C3G diet modulates the H3K4me3 signals within promoter regions, subsequently affecting various signaling pathways, including integrin-linked kinase signaling, which is associated with anti-inflammatory responses [32, 33]. Additionally, plenty of polyphenols were identified as inhibitors of lysine-specific demethylase-1 (LSD-1) inhibitors, a protein that regulates histone methylation [34]. It is plausible that C3G or its metabolites may directly influence histone-modifying enzymes.

Cyanidin-3-O-galactoside (C3Gal) and Cyanidin-3-glucoside (C3G) are two predominant anthocyanins found in Chokeberry. A study investigating the anti-inflammatory effects of chokeberry extract (CBE) on palmitic acid (PA)-induced inflammation in human preadipocyte cells revealed that CBE suppresses PA-induced IL-6 mRNA expression by augmenting DNA methylation of the IL-6 promoter region [35].

Despite numerous clinical studies investigating the effects of TC juice on metabolic syndrome (MetS), including gout [28] obesity [36] and cardiovascular diseases [37] limited research has elucidated the effects of anthocyanins in TC juice on MetS. Metabolic syndrome, such as obesity, is a chronic progression accompanied by a series of oxidative stress and inflammation [38]. Consequently, anthocyanins, renowned for their antioxidant and anti-inflammatory properties, possess the potential to modulate metabolic syndromes.

In the Bhaswant et al. study, Wistar rats were fed a high-fat diet for 16 weeks to induce metabolic syndrome. Subsequently, the rats exhibited various signs of metabolic syndrome, including visceral adiposity, impaired glucose tolerance, hypertension, cardiovascular remodeling, increased collagen deposition in the left ventricle, non-alcoholic fatty liver disease, elevated plasma liver enzymes, and increased inflammatory cell infiltration in the heart and liver. Notably, the addition of C3G to the high-fat diet mitigated the progression of these metabolic syndrome signs. This resulted in a reduction in body weight gain, decreased abdominal fat accumulation, improved lipid profile and glucose metabolism, and enhanced cardiovascular and hepatic structure and function [39].

From the perspective of attenuating obesity, C3G modulates lipid metabolism via diminishing lipid synthesis, boosting fatty acid oxidation, and lessening lipid accumulation. Also, C3G regulates energy metabolism by facilitating energy consumption, inducing brown adipose tissue activities, and activating mitochondrial biogenesis. C3G shows antioxidative capabilities by promoting the expression of antioxidant enzymes, lowering ROS production, and activating the Nrf2/AMPK signaling pathway. The anti-inflammatory mechanisms exhibited by C3G include inhibition of the NF-kB pathway, decreased production of pro-inflammatory cytokines, and modulation of macrophage polarization from the pro-inflammatory M1 phenotype to the anti-inflammatory M2 phenotype. These mechanisms collectively contribute to the mitigation of inflammatory bowel disease [40].

In addition to its impact on metabolic syndromes, C3G exerts neuroprotective effects on the central nervous system, thereby preventing cerebral ischemia, Alzheimer’s disease, Parkinson’s disease, multiple sclerosis, and glioblastoma. The primary protective mechanisms involve the inhibition of oxidative stress and neuroinflammation. Other implicated mechanisms include the suppression of c-Jun N-terminal kinase (JNK) activation, the amelioration of cellular degeneration, the activation of the brain-derived neurotrophic factor (BDNF) signaling pathway, and the restoration of Ca^2+^ and Zn^2+^ homeostasis [41].

Additionally, protocatechuic acid (PCA) is a phenolic acid and a primary metabolite of anthocyanins. PCA also demonstrates its pharmacological activities, including antioxidant, anti-inflammatory, neuroprotective, anticancer, and protection against metabolic syndromes. PCA holds potential as a biomarker and therapeutic agent in the investigation of anthocyanins’ effects [42].

As an essential organelle for eukaryotic cells, mitochondria perform vital function in energy production and metabolic processes. Regular mitochondrial function is critical in cell respiration and ATP generation. Inflammation and oxidative stress like ROS might influence the structure or function of mitochondria, and C3G is a kind of anthocyanin which can suppress the oxidation as well as reduce the inflammatory reaction [43]. In Jinying W. et al. study, HK-2 cells were used to reveal the effect of C3G on suppressing the generation of HG-mediated ROS and the expression of cleaved caspase-3 and the Bax/Bcl-2 ratio. To investigate the protection of C3G from oxidative stress, cells were exposed to HG conditions and checked relevent enzyme genes related to antioxidation. RT-qPCR was performed, and the results showed the Bcl-2/Bax and cleaved caspase-3/caspase-3 ratios were significantly suppressed in the HG + C3G group. Furthermore, the suppression of cyt c expressing caused by HG was attenuated by the treatment with C3G. To test whether C3G is involved in HG-induced apoptosis via p38 MAPK and ERK1/2 associated pathways, researchers measured the expression of p-p38 MAPK and p-ERK1/2. The results suggested the treatment with C3G significantly suppressed the HG-induced increase of p-p38 MAPK and p-ERK1/2 levels, which can reduce HG-induced apoptosis [44]. 

Qiao H. et al. studied the mechanism of C3G regulating mitochondrial damage induced by ethanol. The Gao-binge alcoholic liver disease (ALD) mouse model and AML12 hepatocytes were used, and the regulatory effect and mechanism of C3G on mitochondrial damage were evaluated by Western blot and qPCR analysis. In addition to downregulating the expression of Cytochrome P450 2E1 (CYP2E1), a critical enzyme that catalyzes ethanol oxidation to generate ROS, C3G can also change the morphology of mitochondria and enhance the expression of mitochondrial autophagy-related genes, thereby protecting mitochondria from damage. Transmission electron microscopy showed that the mitochondria of mice in the ALD group were swollen, vacuolated, and some cristae were fragmented. However, mice in C3G-treated group did not show the damaged mitochondria. In addition, mRNA expression of mitophagy-related genes was tested to investigate C3G protecting against ethanol-induced mitochondrial damage. The results showed that in the ALD group, the expression of Ubiquitin-specific protease 30 (Usp30) was reduced, while in the C3G group, the expression was increased. In summary, C3G can reduce mitochondrial-derived ROS in ethanol-damaged cells by regulating mitochondrial autophagy and its related genes [45]. 

Due to their inherent characteristics, anthocyanins and C3G can be effectively utilized as preventive measures against the progression of MetS and cancers developed from elevated oxidative stress and inflammation [23]. Although a wide range of TCJC research has been conducted, the underlying mechanisms of anthocyanins rewiring metabolic pathways and epigenetics that contribute to the prevention and/or treatment of MetS and cancer remain limited. Consequently, this area presents a potential avenue for further research.

### Flavonoids

Polyphenols are a major group of phytochemicals present in TC juice, which include the bioactive ingredients for antioxidation and anti-inflammation like flavonoids, quercetin, kaempferol, and epicatechin. Based on previous studies, flavonoids can interact with ROS/reactive nitrogen species (RNS) and then inhibit the chain reaction before cell viability is impacted [46]. Also, since the bioactive components it has, the polyphenols can decrease the possibility of chronic diseases such as cancer, CVDs, diabetes, and neurodegenerative disorders [47]. 

Flavonoids are one of the main compounds present in TC juice, which include kaempferol, myricetin, fisetin, silymarin, rutin, isorhamnetin, and quercetin [48]. Some other 3-hydroxy derivatives of flavonones like simple monomer catechin, epicatechin, and complex epigallocatechin, procyanidin are also bioactive ingredients [49, 50]. Flavonoids as exogenous antioxidants, can decrease ROS generation and regulate oxidation-relevant enzymes expression to achieve its effects [51, 52]. In Do-Wan et al.‘s study, they found that ROS production induced by fine particulate matter (PM) could be inhibited by TC since the presented quercetin, kaempferol, and chlorogenic acid [53]. PM10 as an air pollutant causes cellular damages by damaging organelles like endoplasmic reticulum (ER), lysosomes, and mitochondria [54]. Flavonoids can inhibit the oxidative keratinocyte apoptosis induced by PM10 through downregulating NF-κB process [53]. Also, previous studies confirm that epigallocatechin gallate works as a phenolic antioxidant can inhibit ROS generation and relevant cytotoxicity [55]. 

Moreover, Shasika et al.‘s study verified the effects of TC in adipose tissue inflammation control with in vivo and in vitro rats’ models. In their results, TC can inhibit the inflammatory response in Zucker fatty rats’ adipose tissue by downregulating the expression of relevant pro-inflammatory biomarkers like TNF-α and IL-6, and inducing the generation of anti-inflammatory markers like NF-κB at the same time [56]. 

### Ascorbic Acid & Beta-Carotene

TC juice concentrate is a rich source of essential micronutrients, particularly vitamin C and beta-carotene, which have been recognized for their significant contributions to both epigenetic modulation and metabolic regulation. These bioactive compounds possess the potential to impact chronic disease prevention through their antioxidative and regulatory roles.

Ascorbic Acid, also known as vitamin C, plays a crucial role as a cofactor for ten-eleven translocation (TET) enzymes, which are responsible for the demethylation of DNA. This process is particularly important for restoring normal gene expression patterns in the context of aberrant DNA hypermethylation often seen in various diseases, including cancer [57, 58]. By facilitating DNA demethylation, vitamin C has been implicated in the reactivation of tumor suppressor genes, thereby contributing to potential anti-cancer effects.

Vitamin C’s ability to reverse aberrant DNA methylation has been shown to restore the activity of key tumor suppressor genes. For example, CDKN1A (p21), a cyclin-dependent kinase inhibitor critical for cell cycle regulation, can be reactivated to inhibit tumor growth. Similarly, MLH1, a mismatch repair gene often silenced by hypermethylation in cancers, can have its function restored, thereby supporting DNA repair mechanisms. Vitamin C has also been reported to influence BRCA1, a gene commonly silenced in breast and ovarian cancers, enhancing its normal tumor-suppressing functions. Additionally, RASSF1A, a gene frequently hypermethylated in various cancers, can be reactivated by vitamin C to inhibit proliferation and promote apoptosis of tumor cells [57–59].

Similarly, beta-carotene impacts histone modifications, particularly histone acetylation and methylation, through its metabolism into retinoic acid. Retinoic acid serves as a signaling molecule that modulates gene transcription by interacting with nuclear receptors, such as retinoic acid receptors (RARs) and retinoid X receptors (RXRs), and chromatin-modifying enzymes, including histone acetyltransferases (HATs) and histone deacetylases (HDACs) [60]. This regulation is pivotal in cellular differentiation and apoptosis, processes that are frequently dysregulated in chronic diseases such as cancer and cardiovascular disorders.

The antioxidative properties of vitamin C and beta-carotene are central to their impact on metabolic signaling pathways. By scavenging reactive oxygen species (ROS), these compounds mitigate oxidative stress, which is a key driver of metabolic dysfunction in chronic diseases like diabetes and cardiovascular disorders [61, 62].

Vitamin C directly influences metabolic pathways by supporting the biosynthesis of carnitine, a molecule essential for the transport of fatty acids into mitochondria for beta-oxidation [63]. This role underscores its importance in maintaining energy homeostasis and preventing lipid accumulation, which are critical in metabolic syndrome. Additionally, vitamin C influences the tricarboxylic acid (TCA) cycle by enhancing the activity of key enzymes such as aconitase, which contributes to efficient energy production [64]. It also plays a role in the pentose phosphate pathway (PPP) by maintaining reduced glutathione levels, ensuring proper cellular redox balance and biosynthesis of nucleotides [65]. Furthermore, vitamin C supports the synthesis of neurotransmitters such as norepinephrine by acting as a cofactor for dopamine beta-hydroxylase, underscoring its importance in metabolic and neuronal health [66].

Beta-carotene is metabolized into retinoic acid, which has been shown to regulate lipid and glucose metabolism. Retinoic acid enhances insulin sensitivity by modulating peroxisome proliferator-activated receptors (PPARs), a group of nuclear receptor proteins that regulate the expression of genes involved in glucose and lipid metabolism [67]. Additionally, beta-carotene’s antioxidative activity contributes to the reduction of inflammation, a key factor in the pathogenesis of metabolic disorders [68].

The combined effects of vitamin C and beta-carotene on epigenetic and metabolic pathways highlight their potential in chronic disease prevention and management. Preclinical studies suggest that supplementation with TC juice concentrate, rich in these compounds, may reduce the risk of diseases such as diabetes, cardiovascular disorders, and certain cancers. These benefits are attributed to its ability to modulate oxidative stress and inflammation [69]restore epigenetic balance, and enhance metabolic efficiency through improved lipid and glucose regulation. Despite promising findings, further research is needed to delineate the precise mechanisms through which vitamin C and beta-carotene exert their effects. Long-term clinical trials focusing on dose-response relationships and molecular pathways will be critical for establishing evidence-based recommendations for TC juice concentrate consumption.

Despite promising findings, further research is needed to delineate the precise mechanisms through which vitamin C and beta-carotene exert their effects. Long-term clinical trials focusing on dose-response relationships and molecular pathways will be critical for establishing evidence-based recommendations for TC juice concentrate consumption.

### Ellagic Acid

Ellagic acid (EA) demonstrates numerous health benefits, such as antibacterial, anti-inflammatory, antihyperglycemic, ant atherosclerotic and antihypertensive properties. Although its positive effects are well-documented, the mechanisms by which EA influences gene expression through epigenetic pathways, particularly via microRNAs (miRNAs) that suppress gene translation, are not fully understood [70]. A succinctly tabulation was formulated in silico setup was captured and indicated that Upregulated and downregulated ncRNAs has been taken place. EA a polyphenolic compound, regulates non-coding RNAs, particularly microRNAs (miRNAs), which are crucial in gene expression [71].

One study demonstrates the epigenetic potential of polyphenols in modulating histone-modifying enzymes. Urolithins B and C showed significant HAT inhibition (> 50%), while ellagitannin oenothein B and gallic acid moderately reduced HAT activity. HDAC activity was unaffected, emphasizing polyphenols’ selective influence on HAT in inflammatory models [72]. EA modulates histone methylation and inhibits CARM1, reducing NF-κB-mediated inflammation and hyperdimethylation of histone 3 arginine 17. It also downregulates PPARγ, impacting metabolic regulation through epigenetic mechanisms, that indicated EA impacted on epigenetics [73]. Ellagic acid inhibits key epigenetic enzymes, EZH2 and PRMT5:MEP50, with strong binding affinities, reducing their catalytic products, H3K27me3 and H4R3me2s [74]. Ellagitannins are metabolized into urolithin A, which enhances O2−-generating activity (~ 175%) in macrophages by upregulating gp91-phox expression through histone acetylation. In contrast, ellagic acid reduces activity (~ 70%) by suppressing gp91-phox expression. This highlights urolithin A’s role in modulating oxidative function via epigenetic mechanisms [75]. It has an antioxidant and epigenetic regulatory properties, including chemopreventive effects. Ellagic acid, isolated from the extract, selectively inhibits PRMT4 (CARM1), reducing H3R17 methylation. It also shows cell-specific effects on p21 expression, suppressing it in certain cell types [76]. In human models, EA increases the expression of tumor-suppressive miRNAs such as let-7a, miR-215, and miR-34c, enhancing anti-tumor activity, apoptosis, and reduced cell migration. It simultaneously suppresses oncogenic miRNAs, including miR-224, miR-29b, and miR-21, thereby reducing pathways that promote cancer. In rat models, EA upregulates tumor-suppressive miRNAs like miR-122 and miR-127 while downregulating oncogenic ones like miR-182 and miR-375 [72]. Ellagic acid demonstrates significant potential in targeting metabolic and inflammatory pathways in cancer therapy. By inhibiting key oncogene activation and tumor suppressor silencing. Metabolically, these actions induce autophagy and apoptosis, while anti-inflammatory effects suppress tumor proliferation.

In vivo studies using mouse xenografts confirmed that oral administration of ellagic acid significantly reduced tumor size and expression of proliferative markers like ki67 [75]. Metabolomics uncovers altered pathways in diseases, such as the Warburg effect in cancer, where mutations in IDH1/2 produce oncometabolites like 2-hydroxyglutarate, disrupting TET enzymes and driving tumor growth. In cardiovascular diseases, markers like succinate, GABA, and TMAO highlight mitochondrial and lipid metabolism dysfunctions. Epigenetic and metabolic biomarkers like 5hmC and lactate enhance diagnostics. Therapies combining DNMT/HDAC inhibitors with metabolic modulators (e.g., IDH inhibitors) show promise, while precision medicine leverages these profiles for targeted treatments [77]. EA mitigates diabetic cardiac dysfunction by enhancing mitochondrial function and modulating DNA 5-hydroxymethylcytosine (5hmC) levels. EA upregulates TET enzyme activity and mitochondrial complexes I/III/V, likely through improvements in the tricarboxylic acid (TCA) cycle. This results in increased 5hmC levels in cardiac DNA, highlighting EA’s potential as a modulator of epigenetic and mitochondrial pathways in diabetes-induced cardiac dysfunction [78]. A study on Polyphenol-rich extracts show ellagic acid improved metabolic health in high-fat diet-fed mice without affecting weight or food intake. Mice showed reduced fasting glucose, improved glucose tolerance, and trends toward better physical performance. Anti-inflammatory effects were observed with lower cytokine levels, alongside increased expression of fatty acid oxidation markers (CPT1-α, ACOX-1). Results suggest that polyphenols enhance metabolism through anti-inflammatory and lipid-modulating mechanisms, potentially via nuclear hormone receptor activation [79]. Ellagic acid lowers blood pressure via enhanced nitric oxide (NO) bioavailability, eNOS activation, and antioxidant defense through the Nrf2/ARE pathway. It inhibits inflammasome activation (e.g., NLRP3) and ACE activity, modulates mitochondrial function, and regulates β-adrenergic signaling, effectively reducing oxidative stress, vascular inflammation, and cardiac remodeling [80].

### Chlorogenic Acid

Chlorogenic Acid (CGA) is an important polyphenol compound in the human diet and is widely found in TC juice and its concentrates. The multifaceted roles of CGA in epigenetic regulation and metabolic modulation have been widely investigated and demonstrated. The effects of CGA on epigenetic mechanisms primarily involve DNA methylation, histone modification, and the modulation of non-coding RNAs. CGA is also closely related to metabolic pathways and has important potential for the prevention and treatment of cancer and metabolic diseases such as diabetes and cardiovascular diseases.

Firstly, CGA can significantly affect the level of DNA methylation, and it shows a broad application prospect in cancer therapy by inhibiting DNA methyltransferases (DNMT), especially DNMT1. CGA inhibited DNMT1 significantly with an IC50 value of 0.9 µM in vitro, which indicates that CGA can effectively inhibit the methylation level of tumor-related genes at a low concentration and restore their normal functions [81]. For example, in HepG2 hepatocellular cancer cells, the action of CGA resulted in the up-regulation of the expression of the tumor suppressor genes p53 and p21, which significantly inhibited cell proliferation, migration and invasion [82]. In addition, CGA is able to activate retinoic acid receptor β (RARβ) gene expression by reducing methylation of its promoter region. This effect was confirmed in breast cancer cells MCF-7 and MDA-MB-231, further demonstrating the central role of CGA in epigenetic regulation [83]. The mechanism is primarily through the increased accumulation of S-adenosyl-L-homocysteine (SAH), a compound that is a noncompetitive inhibitor of DNMT, which inhibits the DNA methylation response [81]. Secondly, the regulation of histone modification by CGA also plays an important anti-cancer role. Studies have shown that CGA is a potent histone deacetylase (HDAC) inhibitor, which can significantly reduce the activities of HDAC-6 and HDAC-8 in non-small cell lung cancer cells [84, 85]. For example, by inhibiting HDAC-6 activity, CGA reduces the expression level of acetylated NF-κB [85]. This regulation is associated with the inhibition of the transcriptional activities of pro-inflammatory factors, such as TNF-α and IL-1β, which in turn reduces the ability of tumors to proliferate and metastasize [86]. In addition, CGA further inhibits tumor growth by regulating the acetylation level of histones, affecting gene expression and cell cycle regulation. For instance, in hepatocellular carcinoma HepG2 cells, CGA was able to prevent the degradation of the extracellular matrix by reducing the expression of MMP-2 and MMP-9, thus inhibiting the migration and invasion of tumor cells [82]. Since liver fibrosis is a precursor lesion of hepatocellular cancer, the regulatory role of CGA is important for liver cancer prevention. Third, CGA has also demonstrated its unique epigenetic effects in the regulation of non-coding RNA. Taking microRNA (miRNA) as an example, CGA can intervene in the transforming growth factor β1 (TGF-β1)/Smad7 signaling pathway by regulating the expression of miR-21, thereby reducing the onset and development of liver fibrosis [87]. In addition, CGA can suppress the proliferation, migration and invasive ability of liver and lung cancer cells by down-regulating the expression of the miR-17 family (including miR-20a, miR-93 and miR-106b), thus further demonstrating the diversity and breadth of its epigenetic regulation [88]. The epigenetic effects of CGA are closely linked to its metabolic regulatory capacity, and its remodeling of multiple metabolic pathways further strengthens its potential for anticancer and disease prevention. In cancer metabolism studies, the mechanism of action of CGA suggests that it can regulate metabolic signaling through multiple pathways. For example, CGA inhibits tumor cell growth in pancreatic cancer (PDAC) therapy by inhibiting the c-Myc-TFR1 axis, interfering with iron metabolism and mitochondrial respiration, and significantly decreasing cellular energy production, as well as decreasing reactive oxygen species (ROS) generation [89]. In addition, CGA prevents tumor angiogenesis and further cancer cell spread by inhibiting the expression of hypoxia-inducible factor 1α (HIF-1α) and vascular endothelial growth factor (VEGF) [90, 91]. This role has been validated in multiple cancer cell models (e.g., DU145 and A549), further illustrating the role of CGA in metabolic regulation and epigenetic linkage [91–93]. 

In chronic disease prevention and treatment, the metabolic modulation effect of CGA further highlights its preventive value. Studies have shown that CGA can regulate glucose and lipid metabolism by activating the adenylate-activated protein kinase (AMPK) pathway, improve insulin sensitivity, and attenuate fatty liver and insulin resistance induced by high-fat diet [90, 94, 95]. For example, in an experimental diabetes model, CGA effectively improved the metabolic status of diabetic mice by promoting GLUT4 translocation and lipocalin expression. CGA also has protective effects on the cardiovascular system [96]. By inhibiting oxidative stress and inflammation, CGA lowers blood pressure, ameliorates atherosclerosis, and reduces the risk of myocardial infarction [90]. The antioxidant and anti-inflammatory properties of CGA underlie its role in epigenetic regulation and the modulation of metabolic pathways. For example, CGA is able to enhance cellular antioxidant enzyme activity through upregulation of nuclear factor E2-related factor 2 (Nrf2), which reduces ROS production and protects cells from oxidative damage [97]. Meanwhile, CGA inhibits the activity of NF-κB and reduces the expression of pro-inflammatory factors to reduce the inflammatory response further. This dual property has shown important protective effects in a variety of chronic diseases and cancers [98]. In preclinical studies, the effects of CGA have been demonstrated in a variety of models. For example, in the HepG2 nude mouse xenograft tumor model of hepatocellular carcinoma, CGA significantly reduced tumor volume and weight, while inhibiting the proliferation and invasive ability of tumor cells [82]. These findings suggest that CGA can effectively inhibit cancer development and progression and improve metabolic disease status through multi-targeted epigenetic regulation and metabolic reprogramming.

## Conclusion

TC has the potential to prevent chronic diseases through its antioxidant and anti-inflammatory effects. The major bioactive compounds of TC, like anthocyanins, flavonoids, vitamins, beta-carotene, ellagic acid, and chlorogenic acid, have been shown to be effective regulators of epigenetic and metabolic pathways. However, the generalizability of findings to human health still remains uncertain since limited clinical data available. The stability and toxicity of TC ingredients are not clear, due to insufficient human clinical studies. Uncertainties exist about how TC bioactives are absorbed, metabolized, and eliminated, which could influence the therapeutic effects. Moreover, many studies are short-term, which limits the insights into long-term effects of TC bioactives on gene regulation and metabolic syndrome. The therapeutic effects for chronic diseases are not always validated in vivo, even though it has been demonstrated in vitro. Some ingredients like polyphenols are absorbed in the upper gastrointestinal (GI) tract, the bioavailability is significantly lower than vitamin antioxidants. Other unabsorbed ingredients might bind to cell wall or tissue and needed to be further metabolized or eliminate by human body, and these pathways need more research.

TC needs long-term clinical studies to extend interventions and design randomized controlled trials with long-term follow-up to assess the sustained effects of TCJC on inflammatory markers and metabolic outcomes. Meanwhile, investigating the dose-response relationships to determine the most effective and safe dosages is also important for gene modulation and metabolic benefits. In future research, one might focus more on changes in DNA methylation, histone modification, and non-coding RNA expression, which are influenced by TC bioactives to relevant metabolic pathways, like AMPK, SIRT1 and NF-κB.

## Data Availability

No datasets were generated or analysed during the current study.

## Abbreviations

TC: Tart cherry
NF- κB: Nuclear factor kappa B
TNF-α: Tumor Necrosis Factor-Alpha
IL-2: Interleukin-2
IL-6: Interleukin-6
CVD: Cardiovascular diseases
ROS: Reactive oxygen species
PRDX2: Peroxiredoxin 2
TLRs: Toll like receptors
LPS: Lipopolysaccharide
IL-1β: Interleukin-1 beta
COX-2: Cyclooxygenase-2
MAPK: Mitogen-activated protein kinase
AP-1: Activator protein 1
LOX: Lipoxygenase
iNOS: inducible nitric oxide synthase
GPx: Glutathione peroxidase
SOD: Superoxide dismutase
CAT: Catalase
HO-1: Heme oxygenase-1
GSH: Glutathione
AhR: Aromatic hydrocarbon receptor
βCAR: β-carotene
BCO1: Beta-carotene oxygenase 1
RAR: Retinoic acid receptor
RXR: Retinoid X receptor
PPARs: Peroxisome proliferator-actived receptors
Nrf2: Nuclear factor E2-related factor 2
CAT: Catalase
SOD: Superoxide dismutase
EA: Ellagic acid
Bcl-2: B cell lymphoma-2
CGA: Chlorogenic acid
IL-8: Interleukin-8
C3GR: Cyanidin-3-glucosyl-rutinoside
C3R: Cyanidin-3-rutinoside
H3K4me3: tri-methylation of lysine 4 on histone H3
LSD-1: Lysine-specific demethylase-1
C3Gal: Cyanidin-3-O-galactoside
CBE: Chokeberry extract
PA: Palmitic acid
MetS: Metabolic syndrome
AMPK: AMP-activated protein kinase
JNK: c-Jun N-terminal kinase
BDNF: Brain-derived neurotrophic factor
PCA: Protocatechuic acid
TCJC: Tart cherry juice concentrate
RNS: Reactive nitrogen species
PM: Particulate matter
ER: Endoplasmic reticulum
TET: Ten-eleven translocation
CDKN1A: Cyclin Dependent Kinase Inhibitor 1 A
MLH1: MutL homolog 1
BRCA1: Breast Cancer Gene 1
RASSF1A: Ras association domain family 1 isoform A
HATs: Histone acetyltransferases
HDACs: Histone deacetylases
TCA: Tricarboxylic acid
PPP: Pentose phosphate pathway
miRNAs: microRNAs
CARM1: Coactivator-Associated Arginine Methyltransferase 1
EZH2: Enhancer of Zeste Homolog 2
PRMT5: Protein arginine methyltransferase 5
MEP50: Methylosome protein 50
H3K27me3: trimethylation of lysine 27 on histone H3
H4R3me2s: Histone H4 dimethyl Arg3 symmetric
H3R17: Histone H3 methylated at arginine 17
IDH1/2: Isocitrate dehydrogenase 1 and 2
TET: Ten-eleven translocation methylcytosine dioxygenase
GABA: Gamma-aminobutyric acid
TMAO: Trimethylamine N-Oxide
DNMT: DNA methyltransferase
CPT1-α: Carnitine palmitoyl-transferase 1alpha
ACOX-1: Acyl-CoA Oxidase 1
NO: Nitric oxide
eNOS: endothelial nitric oxide synthase
ARE: Antioxidant response element
NLRP3: Nod-like Receptor Protein 3
ACE: Angiotensin-converting enzyme
IC50: Half-maximal inhibitory concentration
HepG2: Hepatoblastoma cell line
MCF-7: Michigan Cancer Foundation-7
MDA-MB-231: Breast cancer cell lines
SAH: S-adenosyl-L-homocysteine
MMP-2: Matrix Metalloproteinase-2
MMP-9: Matrix Metalloproteinase-9
TGF-β1: Transforming growth factor β1
Smad7: Mothers against decapentaplegic homolog 7
PDAC: Pancreatic cancer
TFR1: Transferrin receptor 1
HIF-1α: Hypoxia-inducible factor 1α
VEGF: Vascular endothelial growth factor
DU145: Prostatic carcinoma cell line
A549: Lung carcinoma epithelial cells
GLUT4: Glucose Transporter 4
GI: Gastrointestinal
SIRT1: Silent information regulator sirtuin 1
PCT: Parallel controlled trial
RCT: Randomized controlled trial
CRP: C-reactive protein
BP: Blood pressure
LDL: Low-density lipoprotein
ADA: Adenosine deaminase
MTCJ: Montmorency tart cherry juice
PLA: Placebo
MetS: Metabolic Syndrome
HDL: High-density lipoprotein
HG: High glucose
C3G: Cyanidin-3-O-glucoside
ALD: Alcoholic liver disease
CYP2E1: Cytochrome P450 2E1
Usp30: Ubiquitin-specific protease 30
